# Histomorphometry and Immunohistochemistry of Xenogeneic Collagen Matrix: A Randomized Split‐Mouth Clinical Study

**DOI:** 10.1002/cre2.70494

**Published:** 2026-10-29

**Authors:** Marcela Sandy Valença de Barros, Marcelo Pereira Nunes, Arthur Tenório Ribeiro Clark, Roberto Carlos Mourão Pinho, Gustavo Pina Godoy, Cauê Fontan, Bruna Carvalho de Farias Vajgel, Renata Cimões

**Affiliations:** ^1^ Department of Oral and Maxillofacial Surgery Federal University of Pernambuco Recife Pernambuco Brazil

**Keywords:** collagen matrix, dental implants, soft tissue, subepithelial connective tissue graft

## Abstract

**Objectives:**

To histomorphometrically evaluate collagen formation and immunohistochemically assess soft tissue vascularization around implants after treatment with xenogeneic acellular collagen matrix versus connective tissue graft in a randomized split‐mouth clinical study.

**Materials and Methods:**

Nine patients underwent implant rehabilitation with connective tissue graft and xenogeneic acellular collagen matrix in contralateral edentulous areas. A total of 24 grafts were performed (12 each). Grafts were assigned to healing times of 30 (*n* = 4), 90 (*n* = 4), or 180 days (*n* = 4), at which time samples were collected for histological and immunohistochemical analysis. Collagen quality and density were assessed histologically. Vascularization, vessel density, and diameter were evaluated immunohistochemically. Samples were stained with Masson's trichrome for collagen and immunostained with CD34 for vascular endothelium; the grafted site was the unit of analysis.

**Results:**

The percentage of collagen was higher in sites biopsied at 180 days for both grafts and was similar between groups at all healing periods. Xenogeneic acellular collagen matrix showed vascularization patterns similar to autogenous connective tissue graft, with higher vessel density and vascular percentage at 90 days, while differences between groups were observed only at 30 days.

**Conclusions:**

Within the limitations of this study, xenogeneic acellular collagen matrix demonstrated collagen maturation at the later healing periods and vascularization patterns with histological characteristics similar to those of the connective tissue graft during the evaluated healing periods.

**Trial Registration:** Brazilian Registry of Clinical Trials (ReBEC), RBR‐4vgzyt.

## 1 Introduction

Esthetic and functional rehabilitation with dental implants and the maintenance of a gingival architecture that harmonizes with the adjacent dentition are a challenge in dentistry. After extraction, approximately 2–4 mm of vestibular bone is lost, making it necessary to correct bone defects and to reconstruct the peri‐implant soft tissue architecture (Buser et al. 2011; Lekovic et al. 1997). The subepithelial connective tissue graft (CTG) is considered the gold standard for soft tissue thickening and volume gain. In addition, there is evidence that the thickness of the vertical mucosal tissue is an important factor in bone stability, implant longevity, and prevention of peri‐implant tissue recession, which usually accompany alveolar crestal bone loss (Puisys and Linkevicius 2015).

However, the additional morbidity caused by the palatal CTG harvesting technique is a major disadvantage of the procedure because it is correlated with patient morbidity, graft quality, and poor bleeding control (Tavelli et al. 2020). The need for a second surgical harvesting site increases the duration of the procedure. Additionally, the limited availability of connective tissue makes it impossible to treat several peri‐implant beds.

Allogeneic alternatives, such as the acellular dermal matrix (ADM), have also been proposed; however, as they are derived from human tissue, they carry a potential risk of disease transmission, and their clinical use is restricted in most countries (Pabst et al. 2014).

To overcome the disadvantages associated with CTG and ADM, collagen‐based xenogeneic materials have been developed in recent years to promote keratinized tissue regeneration and for root coverage procedures. One of these materials is xenogeneic acellular collagen matrix (XCM) derived from porcine dermis, which consists of collagen and elastin with a three‐dimensional (3D) structure of natural collagen that resembles human connective tissue (Ramachandra et al. 2014). XCM undergoes a multistep purification process to remove all antigenic components so that the 3D matrix will promote the proliferation of fibroblasts and endothelial cells, facilitating rapid revascularization of its structure (Rothamel et al. 2014) and subsequent integration of soft tissues (Kasaj et al. 2016).

One of the biological factors associated with graft integration is the vascularization of the implanted tissue or matrix. This process contributes to nutrient delivery and tissue remodeling during healing. Delayed vascularization has been described as a potential limitation of artificial CTG substitutes due to the absence of intrinsic vasculature (Supp et al. 2000). Clinical and histological studies have been conducted since 1970 to explain the mechanisms of CTG revascularization and integration to the recipient bed, as well as factors that may influence its success or failure, which include thickness, (Soehren et al. 1973) the amount of connective tissue, and the nature of the receptor site (periosteum or bare bone) (James and McFall 1978; Mormann et al. 1981).

Recent studies have proposed XCM as a potential alternative to CTG due to its biomechanical and biochemical properties (Puisys et al. 2019). Histological data have suggested favorable integration of XCM into host tissues, with no signs of adverse reactions or a significant inflammatory response. In addition, XCM has advantages such as avoiding a second surgical site for graft harvesting, reduction of operative time, the absence of human donors, and no limits on the size of the surgical site (Bottino et al. 2009; Kasaj et al. 2016). Although XCM has already been proposed as an alternative to autologous grafts, histological comparisons between XCM and CTG are absent from the literature (Tavelli et al. 2020). It is therefore important to compare the histological and immunohistochemical characteristics observed after the use of XCM and CTG in peri‐implant soft tissue augmentation procedures. Thus, the objective of this study was to histomorphometrically evaluate collagen formation and immunohistochemically assess soft tissue vascularization around implants after treatment with XCM compared to CTG through a randomized split‐mouth clinical study.

## 2 Materials and Methods

This randomized split‐mouth clinical study followed the CONSORT Group guidelines (Hopewell et al. 2025), was registered on the website of the Brazilian Registry of Clinical Trials (*Registro Brasileiro de Ensaios Clínicos*) under ID No. RBR‐4vgzyt and was approved by the Research Ethics Committee of the Health Sciences Center of Federal University of Pernambuco (*Universidade Federal de Pernambuco* – UFPE) under No. 2,219,548.

The registered public and scientific titles and the description of the interventions correspond to a randomized split‐mouth design, in which each participant received both interventions in contralateral sites and served as their own control; however, the study design field of the registration form was recorded as parallel‐arm, which does not reflect the design planned and conducted. The registered target sample size was 16 participants; nine participants were enrolled. The registered primary outcome (clinical assessment of peri‐implant soft tissue thickness) is outside the scope of the present report, and this article presents the histomorphometric and immunohistochemical outcomes of the same registered trial.

The primary outcome was collagen formation assessed by histomorphometric analysis. Secondary outcomes included vessel density, vessel diameter, and percentage of vascularization assessed by immunohistochemistry. Partially edentulous patients who required dental implants and grafting of gingival tissue in contralateral regions, referred for treatment at the Graduate Dentistry Clinic of UFPE, Recife, Brazil, between September 2018 and May 2019, were eligible for the study. The study participants included individuals who met the following inclusion criteria: lack of teeth in the posterior region of the same arch on contralateral sides; minimum bone width of 6 mm; minimum of 2 mm of keratinized gingiva; not needing bone regeneration; healthy soft tissue; good oral hygiene (visible plaque index < 20% and bleeding on probing index < 20%); age between 18 and 59 years; no allergy to collagen; no tobacco use; not pregnant or lactating; and no systemic disorders. All patients who agreed to participate signed an informed consent form after a clear explanation of the procedures.

A total of nine patients participated in the study. All underwent, during a single surgical procedure, the installation of implants and both CTG (control) and XCM graft (test) in contralateral edentulous areas in one or more arches. Of the nine patients, six had only one test area and one control area, while three individuals had two test areas and two control areas, totaling 24 grafted beds (CTG = 12; XCM = 12). The allocation of CTG and XCM sites was performed at the graft‐site level within the split‐mouth design, allowing each participant to serve as their own control. Grafted sites were distributed in contralateral posterior regions of the same arch in order to reduce anatomical variability between treatment sites. The study was not designed to compare outcomes according to maxillary or mandibular location, and the limited sample size did not allow stratified anatomical analyses. Baseline demographic and clinical characteristics of the study sample are presented in Table 1. In the presurgical phase, all patients underwent an oral control and adjustment phase and were then referred to surgery.

**Table 1:** Baseline demographic and clinical characteristics of the study sample.

Variable	Total
Patients	9
Age (years), mean ± SD	44.11 ± 8.60
Sex	5 men/4 women
Grafted sites	24
CTG sites	12
XCM sites	12
Implant diameter	3.3–4.1 mm
Healing periods	30, 90, 180 days
Smoking status	Non‐smokers

Surgery was performed in both groups under local anesthesia (4% articaine with 1:100,000 epinephrine; DFL, Rio de Janeiro, Brazil). An incision was made at the top of the bone crest, and a full‐thickness flap was raised to access the recipient site. All patients were rehabilitated with bone‐level‐tapered (BLT) implants (Straumann Implant System, Basel, Switzerland) with diameters ranging from 3.3 to 4.1 mm and lengths ranging from 6 to 12 mm. For all implants, an implant cover 0.5 mm in height was placed. After implant installation and flap elevation, the recipient sites were allocated to the test group (XCM) and control group (CTG) by a simple draw performed by a team member not involved in the clinical or data collection procedures. The two graft options were placed in separate opaque envelopes, and the draw determined which surgical site received each material (XCM, envelope 1; CTG, envelope 2), ensuring allocation concealment (Figure 1). Because both interventions were performed during the same surgical procedure, patients were blinded to the treatment received at each site.

**Figure 1:**
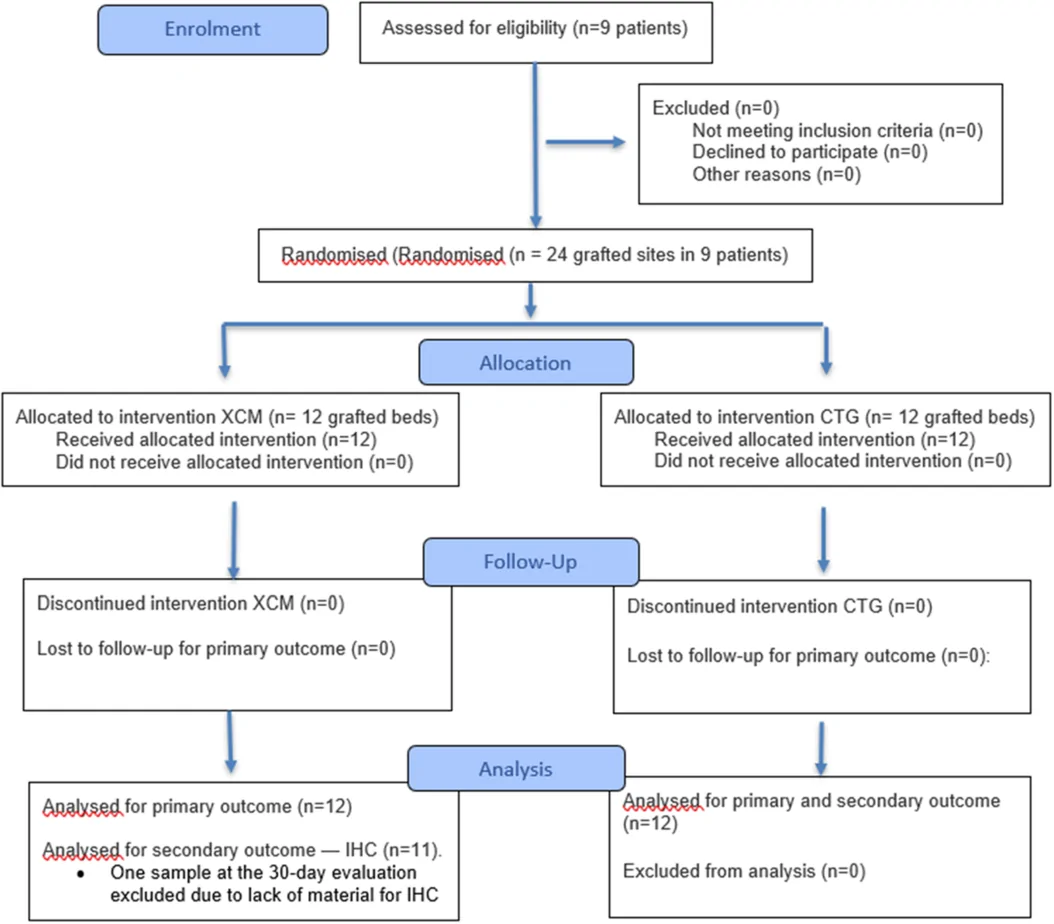
CONSORT flow diagram of the study. The diagram shows the number of patients assessed for eligibility and enrolled, as well as the number of grafted beds randomized, allocated to each intervention (XCM or CTG), followed‐up, and analyzed for primary outcomes.

In the test sites, one standard‐size (15 × 20 mm) XCM graft (1.5–1.9 mm thick) was hydrated with patient blood for 20 min and individually modeled to fit the implant site. The matrix was positioned to cover 10 mm of the buccal surface and 5 mm of the lingual surface beyond the implant margin and to extend to the adjacent teeth. In the control sites (CTG), the graft was harvested from the palate using the free gingival graft technique followed by de‐epithelialization. After fitting both grafts, the sites were sutured to completely cover the implant and graft with single horizontal mattress sutures with 5‐0 thread (PGA Resolon®, Resorba, Nürnberg, Germany).

Patients were instructed not to brush the surgical sites for 2 weeks and to rinse the mouth with 0.12% chlorhexidine digluconate (Colgate, Brazil) twice a day for 2 weeks. The following medications were prescribed: amoxicillin 500 mg; dexamethasone 8 mg; and dipyrone 1 g or paracetamol 750 mg (Ache Laboratórios Farmacêuticos SA, Guarulhos, Brazil). The sutures were removed 15 days after surgery.

After surgery, patients were randomly assigned to one of three healing periods, as described in the registered protocol: 30 days (*n* = 3 patients; 4 XCM and 4 CTG sites), 90 days (*n* = 2 patients; 4 XCM and 4 CTG sites), and 180 days (*n* = 4 patients; 4 XCM and 4 CTG sites). At the predetermined healing time, gingival tissue samples were collected during the implant reopening surgery using a disposable 4‐mm diameter punch (Kolplast, Itupeva, Brazil) under local anesthesia and processed for histomorphometric and immunohistochemical (IHC) analysis. The collected specimens were immediately fixed in 10% neutral buffered formalin.

After tissue removal, all implants received a corresponding healing abutment, and after 6 months, they were rehabilitated with metal‐ceramic crowns.

The fixed samples were dehydrated and embedded in paraffin in the cervicoapical direction to allow the block to be sectioned as parallel to the surface plane as possible. Serial 5‐μm sections were obtained and stained with hematoxylin‐eosin and Masson's trichrome (MT). The images were obtained for analysis by whole‐slide scanning of the histological slides with an automated digital scanner (Pannoramic MIDI II®, 3DHISTECH, Budapest, Hungary). After the scanning step, the slides were evaluated with the aid of image analysis software (Pannoramic Viewer® 1.15.4 RTM, 3DHISTECH, Budapest, Hungary). Based on the scanned images, five fields were randomly selected from each sample, with a final magnification of 200×.

An experienced examiner evaluated the collagen density by classifying the distribution of collagen fibers in the total area as dense or loose. The examiner, an oral pathology specialist, was blinded to the graft type allocated to each site (test or control); blinding to the healing period was not applied, as each participant was scheduled for biopsy at a single predefined time point. To quantify the collagen area, the mean collagen area per field captured on the histological slide (total field area = 683.00 × 352.5 = 240,757.5 μm^2^ [0.24 mm^2^]) was used. The percentage of collagen area per field was calculated with ImageJ® 1.47 (Image Processing and Analysis in Java, National Institute of Mental Health, Bethesda, Maryland, USA) using the color threshold tool in the RGB color function for blue (minimum of 110 and maximum of 191), saturation (minimum of 0 and maximum of 255), and brightness (minimum of 0 and maximum of 255). The data obtained are expressed as the percentage of collagen area relative to the total area of the image (μm^2^).

For the detection of blood vessels in the matrix and surrounding connective tissue, immunohistochemical analysis was performed. The samples were sliced into 4‐μm‐thick sections, spread on silanized glass slides (StarFrost adhesive slides, Knittel Gläser, Braunschweig, Germany), and stained using an anti‐CD34 monoclonal antibody (Dako, Agilent). The Dako REAL EnVision detection system (Agilent) was used to visualize the antibody.

To evaluate vascularization, five fields (200×) were randomly selected per XCM or CTG sample. Images of the histological sections were captured using a workstation comprising a Moticam 1000 1.3‐megapixel video camera system with 1280 × 1024 resolution (Moticam®) coupled to an optical microscope (Labomed Lx 400) and a computer running the Motic Image Plus® 2.0 software. Next, ImageJ® was used to measure the vascularization parameters at the implantation site.

The morphometric parameter adopted was the distribution of the mean area per field captured on the histological slide (total field area = 481.88 × 361.41 = 174,156.25 μm^2^ [0.17 mm^2^]). Based on these data, the diameter of the vessels, their density, and the percentage of vascularization were calculated at each healing period (30, 90, and 180 days). The percentage of vascularization was calculated as the total area of vessels divided by the total area of the image (μm^2^). The mean vessel diameter was determined from three random measurements performed on the lumen of the visible vessels, excluding the longest axis when the vessel was sectioned on the longitudinal axis. Vessel density was determined by counting and is expressed as the number of vessels per square millimeter (vessels/mm^2^).

Data were analyzed descriptively. Owing to the split‐mouth design, XCM and CTG sites within the same participant are paired rather than independent. Considering the limited number of participants (*n* = 9 patients; 12 sites per group distributed across three healing periods), no inferential testing was performed. Data are reported as mean ± standard deviation for each group and healing period to allow comparison with previous histological studies in the field, and differences between groups and across healing periods are described in terms of the observed patterns and the magnitude of the differences. The grafted site was considered the unit of analysis. Because some participants contributed more than one pair of grafted sites, potential clustering effects related to the split‐mouth design cannot be completely excluded.

## 3 Results

The percentage of collagen was higher in sites biopsied at 180 days for both XCM (83.85 ± 9.00%) and CTG (86.71 ± 5.53%) than at the earlier healing periods, and was similar between the groups at all healing periods (Table 2).

**Table 2:** Descriptive measures for the CTG and XCM groups regarding the amount of collagen.

Group	Period (days)	*N*	Mean ± standard deviation
CTG	30	4	59.41 ± 16.68
	90	4	64.75 ± 16.31
	180	4	86.71 ± 5.53
XCM	30	4	52.95 ± 13.30
	90	4	55.73 ± 13.98
	180	4	83.85 ± 9.00

The XCM group had a loose connective tissue pattern at 30 days. In the interval between 90 and 180 days, similar histological characteristics were identified between the XCM and CTG groups. The connective tissue exhibited dense collagen fiber bundles, with uniform distribution at all tissue sites at 180 days, which revealed collagen maturation in both groups (Table 3 and Figure 2).

**Table 3:** Descriptive measures for the CTG and XCM groups regarding the amount and density of collagen.

CTG					XCM			
Period (days)	*N*	Amount of collagen (mean ± SD)	Loose (%)	Dense (%)	*N*	Amount of collagen (mean ± SD)	Loose (%)	Dense (%)
30	4	59.41 ± 16.68	25	75	4	52.95 ± 13.30	100	0
90	4	64.75 ± 16.31	0	100	4	55.73 ± 13.98	25	75
180	4	86.71 ± 5.53	0	100	4	83.85 ± 9.00	0	100

**Figure 2:**
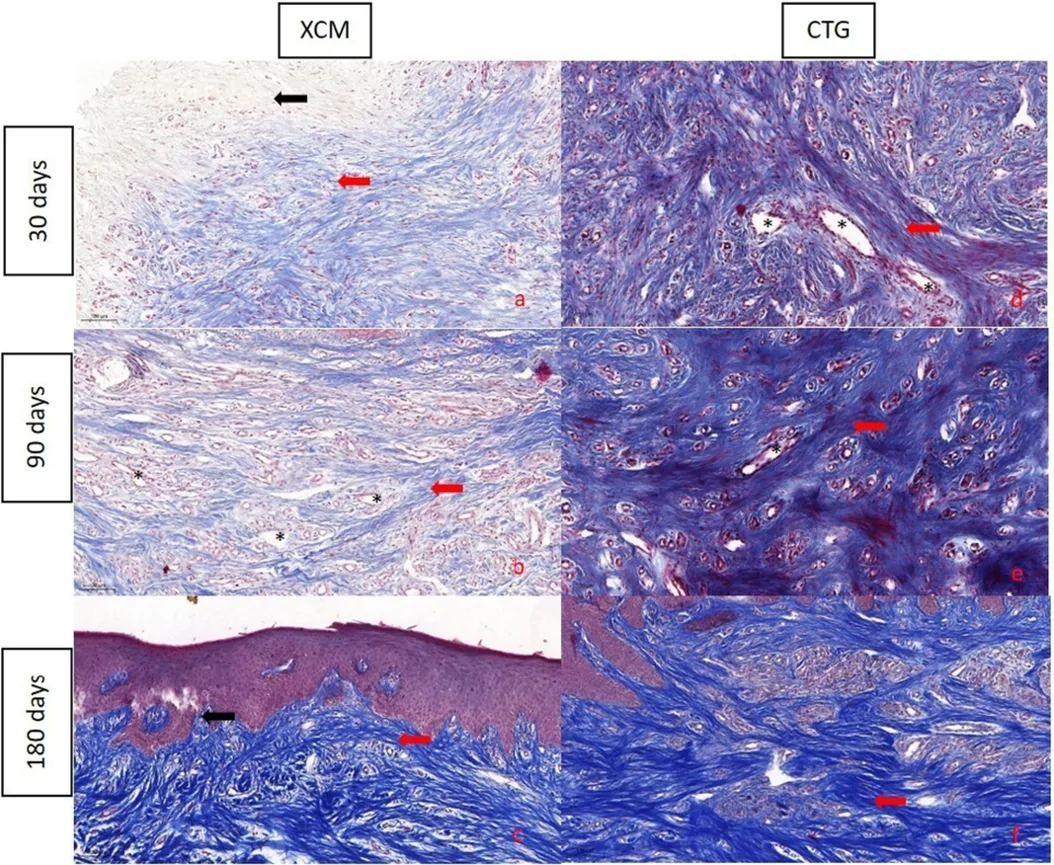
Histological image of the CTG and XCM specimens at 30, 90, and 180 days. (a) Overview of one side of the implanted XCM graft at 30 days (black arrow: lamina propria) (red arrow: XCM). (b) Dense collagen fibers in the area suggestive of XCM traces at 90 days (*vessels). (c) Histological appearance compatible with integration of the XCM graft with the lamina propria at 180 days (red arrow: dense collagen fiber bundles) (* vessels). (d, e, f) Dense collagen fibers at 30, 90, and 180 days in the CTG group, exhibiting greater collagen formation at the later healing periods (MT staining, 10× magnification, scale bar = 100 μm).

One sample from the XCM group was excluded from the 30‐day evaluation due to a lack of material to perform the IHC analysis. Regarding vascularization, the specimens of both groups exhibited blood vessels with varied diameters within the same healing period, and the mean vessel diameters were similar for the CTG group (94.89 ± 20.17 μm) and the XCM group (94.61 ± 5.03 μm) at the 30‐day evaluation. At the 180‐day evaluation, the findings were similar between the CTG (96.01 ± 27.98) and XCM (91.08 ± 30.44) groups. The largest mean vessel diameters in both groups were observed at the 90‐day evaluation (CTG: 118.51 ± 19.01 μm; XCM: 171.09 ± 108.07 μm) (Table 4).

**Table 4:** Descriptive measures for the CTG and XCM groups regarding vascularization.

	Period (days)	*N*	Mean ± standard deviation	*N*	Mean ± standard deviation
Diameter	30	4	94.89 ± 20.17	3	94.61 ± 5.03
	90	4	118.51 ± 19.01	4	171.09 ± 108.07
	180	4	96.01 ± 27.98	4	91.08 ± 30.44
Density	30	4	6.34 ± 5.01	3	5.32 ± 0.76
	90	4	7.12 ± 3.96	4	10.14 ± 1.52
	180	4	2.93 ± 1.76	4	4.69 ± 1.31
Percentage of vascularization (%)	30	4	8.46 ± 1.18	3	11.17 ± 0.24
	90	4	18.71 ± 18.97	4	27.13 ± 10.26
	180	4	8.22 ± 11.89	4	4.62 ± 1.26

Immunohistochemical evaluation of positive vessels showed the highest vessel density at the 90‐day evaluation for both the XCM (10.14 ± 1.52 vessels/mm^2^) and CTG (7.12 ± 3.96 vessels/mm^2^) groups (Table 4). Areas suggestive of XCM traces showed connective tissue permeated by many blood capillaries, indicating active angiogenic activity and tissue integration during the healing process (Figure 3). Nevertheless, the descriptive nature of the present analysis means that the findings should not be interpreted as evidence of clinical equivalence or non‐inferiority, particularly considering the exploratory nature of the study and the limited sample size.

**Figure 3:**
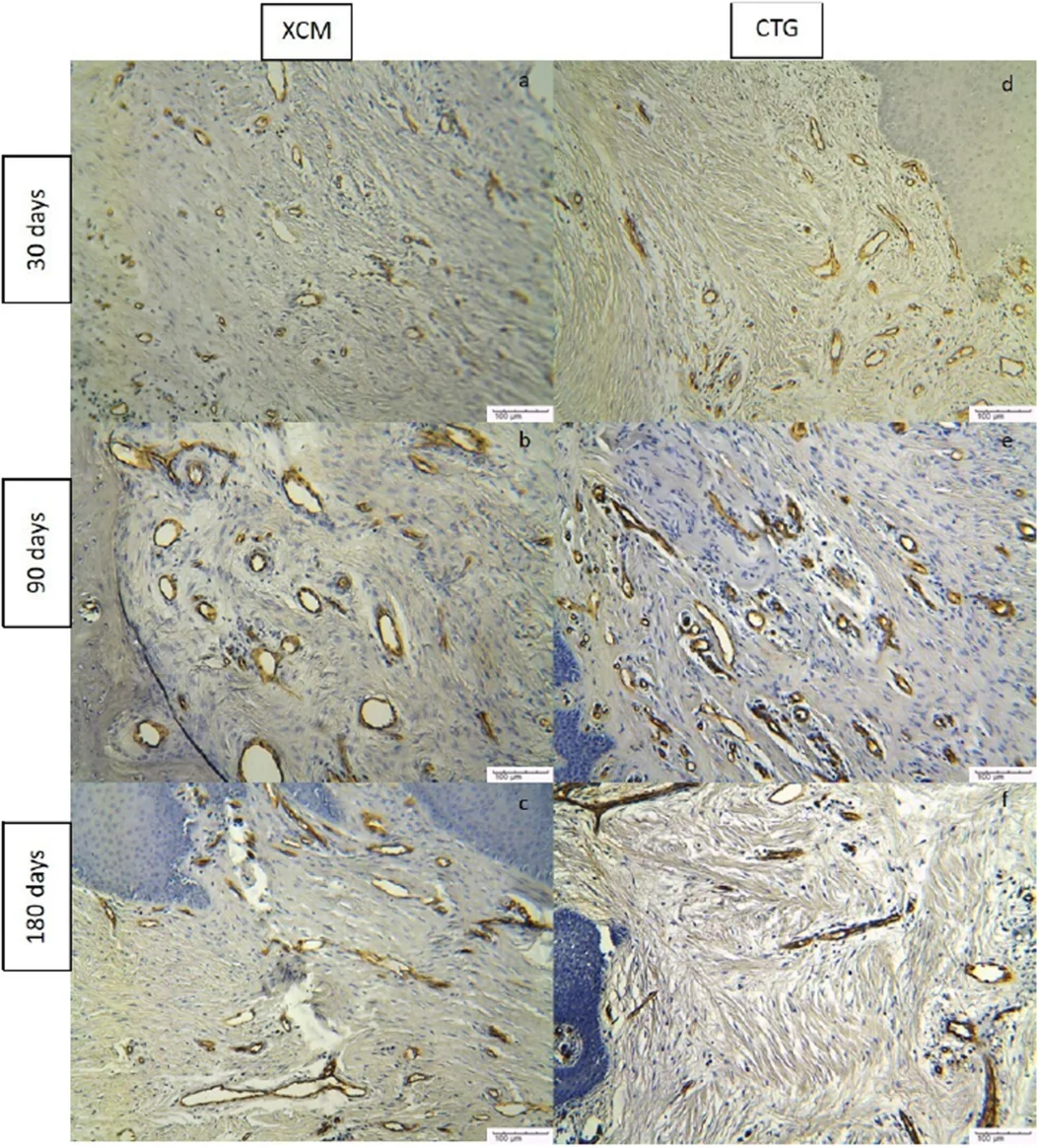
Immunohistochemical images of CD34 antibody immunostaining in CTG and XCM specimens at 30, 90, and 180 days. (a) Overview of one side of the XCM graft exhibiting vascularization at 30 days. (b) Overview of one side of the XCM graft at 90 days, showing numerous blood vessels compared to the first evaluation period. (c) Histological section of the XCM graft at 180 days showing mature vascular structures. (d) Immunohistochemical image representative of one side of the CTG graft showing vascularization at 30 days with permeation of dense collagen fiber bundles. (e) Overview of one side of the CTG at 90 days showing numerous blood vessels with variable calibers. (f) 180‐day evaluation of the graft showing mature blood vessels (10× magnification, scale bar = 100 μm) (CD34 immunostaining).

Overall, the changes observed in the vascular parameters followed the same pattern for both groups, with higher IHC signal values at 90 days. The percentage of vascularization showed greater variation in the XCM group than in the CTG group across the evaluated periods, and the values differed between the groups only at the 30‐day evaluation (Table 4).

## 4 Discussion

The present study evaluated collagen formation and vascularization around implants after the use of XCM compared to CTG at different healing stages through histomorphometric and immunohistochemical analyses. No adverse clinical reactions occurred, and XCM was well tolerated by the patients. However, the present investigation was designed to evaluate histomorphometric and immunohistochemical outcomes, and therefore, no direct clinical measurements of soft tissue thickness, volumetric alterations, or esthetic outcomes were assessed. After 30, 90, and 180 days of healing, XCM exhibited higher collagen percentages at the later healing periods and histological features compatible with tissue integration, corroborating the findings of Thoma et al. (2016), who evaluated the clinical and histological performance of a cross‐linked collagen matrix compared to CTG in patients requiring soft tissue volume augmentation after single‐tooth implant placement. In that study, biopsies obtained at 90 days exhibited dense collagen fibers without a clear distinction between CTG and the newly formed connective tissue.

The only histological study that has evaluated the performance of XCM in humans was conducted by Puisys et al. (2019) in 20 patients. They evaluated the grafts only after 60 days of healing, and, similar to the present study, the specimens exhibited histological features compatible with tissue integration. This performance may be explained by the methods for processing XCM. The porcine dermis is put through a multistep purification process that removes all antigenic components. This processing results in a stable 3D matrix consisting of collagen and elastin with a natural collagen structure that resembles human connective tissue. After implantation, this collagen network serves as a scaffold for the growth of blood vessels and cells, thus supporting revascularization and tissue integration during healing. Simultaneous matrix degradation and collagen production by adherent fibroblasts contribute to progressive replacement of the matrix by newly formed host tissue, and the absence of cross‐linking offers advantages for tissue integration (Rothamel et al. 2014).

The standardization of the surgical procedures and the random allocation of grafts were intended to improve comparability between the groups. No healing complications occurred in any of the evaluated periods in either group, and the histological findings were compatible with tissue integration of XCM. These findings are consistent with the results of other studies that sought to understand the integration process of collagen matrices used for soft tissue thickening, root coverage procedures, and regeneration of keratinized mucosa (McGuire and Scheyer 2010; Thoma et al. 2011).

Unlike those of Thoma et al. (2016), our XCM samples exhibited a similar distribution of the tissue components at the sites of matrix implantation and in the surrounding connective tissue. Moreover, in the present study, islands rich in blood vessels permeating the inside of the matrix and distributed in the lamina propria were observed, suggestive of active vascular remodeling and tissue healing during the integration process. In addition, the XCM exhibited vessel density and vascularization patterns histologically comparable to CTG, with differences observed only at the 30‐day healing period. To achieve histological characteristics similar to those observed in CTGs, the XCM must allow migration and colonization by progenitor cells so that the newly formed tissues can mix with the collagen fibers of the matrix before the XCM is completely degraded, and this occupation is a function of the increased energy demand for the growth of new cells at the site of tissue regeneration, which is met by the delivery of oxygen and metabolites by blood vessels (Park et al. 2018).

Another important aspect related to the biological behavior of XCM and other xenogeneic grafts is their persistence for a sufficient period to allow cell migration into the collagen matrix and subsequent tissue formation (Rothamel et al. 2014). In this regard, the samples exhibited dense collagen fiber bundles characteristic of the matrix 180 days after the procedure, which may indicate persistence of matrix‐related collagen structures during the evaluated healing period. The same was observed by Puisys et al. (2019) at only 60 days of healing. In addition, the samples exhibited loose collagen fibers permeating the dense matrix fibers, indicative of newly formed connective tissue, suggesting ongoing tissue remodeling and integration with the surrounding soft tissue. This conclusion is further substantiated by the higher amount of collagen formed and the greater fiber integration with the lamina propria observed in the sites biopsied at the later healing periods. The CTG samples also exhibited dense collagen formation at the later healing periods, and at the most advanced healing stage, both groups demonstrated comparable collagen deposition patterns from a histomorphometric perspective. These findings differ from those reported by Thoma et al. (2016), who observed a relatively loose connective tissue network in CTG biopsies at 90 days. However, methodological differences between studies, as well as the limited sample size of the present investigation, should be considered when interpreting these divergences.

Thoma et al. (2016) also found a high number of small‐caliber blood vessels at 90 days in CTG samples. However, the samples were evaluated by visual inspection and not by objective criteria as in the present study. In addition, our findings were partially divergent. At 90 days, the immunohistochemical evaluation of XCM showed the highest values of vessel diameter, vessel density, and percentage of vascularization among the evaluated periods, suggesting increased angiogenic activity at this intermediate healing stage. These findings are compatible with the healing stages the grafts are in at those times and are consistent with the natural repair process, which involves the regression of inflammatory activity because, as the tissue matures, the endothelial cells undergo programmed cell death (apoptosis) and the number of vascular units is reduced (Lang and Lindhe 2015). In addition, the CTG specimens exhibited the same pattern of changes in vascular parameters as observed in the XCM group.

The present study has limitations that should be considered when interpreting the findings. The sample size was limited, which restricts the precision of the estimates and the ability to detect differences between groups. No formal a priori sample size calculation was performed, and the study should be interpreted as an exploratory histological comparison between the two graft materials. In addition, the healing periods were evaluated cross‐sectionally rather than longitudinally, and no non‐grafted control group was included. Furthermore, the study evaluated only histomorphometric and immunohistochemical outcomes, without assessment of clinical soft tissue thickness, volumetric changes, or long‐term peri‐implant stability.

Moreover, the procedures were performed under favorable clinical conditions in systemically healthy nonsmokers, which may limit the generalizability of the findings. Also, intra‐examiner reproducibility of the histomorphometric measurements and of the collagen density classification was not formally assessed (e.g., intraclass correlation coefficient or kappa statistics), which should be considered when interpreting the present findings. Additionally, complete specifications of the immunohistochemical protocol, including antibody clone and dilution and antigen retrieval and blocking conditions, were not fully documented, which limits the reproducibility of this technique. Therefore, further longitudinal studies with larger samples and clinical outcome measurements are necessary.

Within the limitations of this study, XCM exhibited collagen maturation at the later healing periods and vascularization patterns with histological characteristics similar to those observed in CTG during the evaluated healing periods. However, these findings should be interpreted cautiously, since the present study did not evaluate clinical soft tissue outcomes or long‐term peri‐implant tissue stability.

## Author Contributions

Conceptualization: Renata Cimões, Bruna Carvalho de Farias Vajgel, and Marcelo Pereira Nunes. Methodology: Renata Cimões, Bruna Carvalho de Farias Vajgel, Gustavo Pina Godoy, and Marcela Sandy Valença de Barros. Investigation: Marcela Sandy Valença de Barros, Marcelo Pereira Nunes, Arthur Tenório Ribeiro Clark, and Roberto Carlos Mourão Pinho. Formal Analysis: Gustavo Pina Godoy, Cauê Fontan, and Arthur Tenório Ribeiro Clark. Project Administration: Renata Cimões and Bruna Carvalho de Farias Vajgel. Writing – Original Draft: Marcela Sandy Valença de Barros and Cauê Fontan. Writing – Review and Editing: Renata Cimões, Bruna Carvalho de Farias Vajgel, Gustavo Pina Godoy, and Cauê Fontan.

## Funding

The authors have nothing to report.

## Consent

All participants provided written informed consent prior to inclusion in the study.

## Conflicts of Interest

The authors declare that the company Straumann provided the Mucoderm® material used in this study free of charge. The company had no role in the study design, data collection and analysis, decision to publish, or preparation of the manuscript.

## Data Availability

The data that support the findings of this study are available from the corresponding author upon reasonable request.
